# Personal PM_2.5_ Exposure and Associated Factors Among Adults with Allergic Diseases in an Urban Environment: A Panel Study

**DOI:** 10.3390/toxics13040317

**Published:** 2025-04-18

**Authors:** Shin-Young Park, Hyeok Jang, Jaymin Kwon, Chan-Mi Park, Cheol-Min Lee, Dae-Jin Song

**Affiliations:** 1 Department of Environmental & Chemical Engineering, Seokyeong University, Seoul 02713, Republic of Korea; tlsdud060900@skuniv.ac.kr (S.-Y.P.); amer1can@skuniv.ac.kr (H.J.); 2 Department of Public Health, California State University, Fresno, CA 93740, USA; jkwon@mail.fresnostate.edu; 3 Biomedical Research Center, Korea University Guro Hospital, Seoul 08308, Republic of Korea; chanmipark@korea.ac.kr; 4 Department of Pediatrics, Korea University Guro Hospital, Seoul 08308, Republic of Korea

**Keywords:** personal exposure, PM_2.5_, linear mixed-effects model, outdoor air quality, air purifier

## Abstract

This study analyzed the factors influencing personal PM_2.5_ exposure levels among adults with allergic diseases in Seoul using a linear mixed-effects (LMEs) model. The average personal PM_2.5_ exposure concentration of the study participants was 17.38 μg/m^3^, exceeding the World Health Organization (WHO) daily recommended guideline (15.00 μg/m^3^), though it was relatively low compared to global levels. Inter-individual exposure variability was approximately 43.5%, with exposure levels varying significantly depending on microenvironments. Notably, 58% of participants exhibited higher exposure on weekends compared to weekdays, likely associated with increased outdoor activities. The LMEs model results identified smoking (90.81% higher in smokers), temperature, relative humidity, outdoor pollutants (PM_2.5_, O_3_, CO), indoor PM_2.5_ and CO concentrations, and time spent in residential environments as factors increasing exposure, while rainfall (91.23% reduction), wind speed, and air purifier use were identified as factors reducing exposure. These findings suggest that individual activity patterns and environmental factors significantly influence exposure levels, highlighting the need for personalized mitigation strategies and national fine dust policies. This study is expected to provide scientific evidence contributing to the reduction in health risks and improvement of quality of life for individuals with allergic diseases.

## 1. Introduction

PM_2.5_, with an aerodynamic diameter of 2.5 μm or less, has been identified as a major risk factor for various diseases, including acute and chronic respiratory diseases, cancer, and cardiovascular diseases, through numerous epidemiological studies [1,2,3], and it has also been reported to influence mortality rates [4,5]. Therefore, reducing PM_2.5_ exposure levels is essential to minimize the associated health risks, necessitating continuous monitoring of PM_2.5_ concentrations and prior research to identify factors affecting changes in PM_2.5_ exposure level.

Historically, studies on PM_2.5_ exposure assessment and health impacts have predominantly focused on outdoor PM_2.5_ [6,7,8]. However, as modern individuals spend most of their time in various indoor environments, the correlation between nearby outdoor PM_2.5_ concentrations and actual personal exposure levels is reported to be low [9,10]. Consequently, recent studies have shifted toward directly measuring personal exposure concentrations in diverse population groups to explore their associations with diseases and the factors contributing to increased personal exposure levels [11,12]. Personal exposure concentrations can be influenced by various factors in the surrounding environment where individuals are active, such as smoking, activity duration, and indoor pollutant concentrations [13]. Personal PM_2.5_ exposure levels vary depending on the environmental characteristics of residential spaces and lifestyle patterns, with notably high PM_2.5_ exposure levels observed in microenvironments such as restaurants or bars during activities like cooking and smoking.

Modern individuals exhibit significant differences in activity ranges depending not only on the characteristics of their residential spaces but also on factors such as age, gender, and residential area. For this reason, personal exposure is highly sensitive to individual activity patterns and inter-individual variability [14,15,16]. Given the diversity of environments and the complex influence of personal factors, generalizing the factors affecting personal exposure levels presents limitations, underscoring the need for personalized management strategies. In particular, for allergic diseases such as asthma and rhinitis, increased PM_2.5_ exposure levels are known to elevate symptom prevalence [17,18,19]. For populations vulnerable to PM_2.5_ exposure, such as these, providing tailored strategies is necessary to minimize symptom manifestation.

Previous studies have investigated personal PM_2.5_ exposure using linear mixed-effects models (LMEs) and similar statistical approaches to examine influencing factors. For instance, studies by Chen et al. [20], McCraken et al. [21], and Chen et al. [22] conducted short-term monitoring (approximately 72 and 24 h) to evaluate personal exposure among people, primarily utilizing stationary measurement devices or portable monitors for limited periods. While these studies provided valuable insights into exposure levels and influencing factors, they were limited in capturing long-term variations due to short monitoring durations.

This study aims to evaluate the factors influencing personal PM_2.5_ exposure among adults with allergic diseases (asthma, rhinitis, conjunctivitis) in Seoul, a representative urban area in South Korea. To achieve this objective, PM_2.5_ was continuously measured in real time over two separate monitoring periods using low-cost sensor devices: from November 2022 to April 2023, and from November 2023 to April 2024. These periods include the spring and winter seasons when PM_2.5_ concentrations are typically high in South Korea. This long-term monitoring approach provides more comprehensive and robust insights compared to short-term measurement studies. In addition to real-time PM_2.5_ measurements, three types of data were collected to comprehensively assess personal exposure: Global Positioning System (GPS) coordinates to track participants’ movements, time activity diaries (TAD) to record individual activity patterns, and daily questionnaires to identify microenvironment-specific factors that may affect PM_2.5_ exposure. By integrating these diverse data sources, we aim to utilize high-resolution, long-term data to conduct a more detailed analysis of how environmental and behavioral factors influence prolonged exposure.

## 2. Materials and Methods

### 2.1. Study Design

This study is part of a broader investigation into the association between allergic diseases (asthma, rhinitis, conjunctivitis, and atopic conditions) and exposure to environmental hazards. Among a panel of 142 participants with allergic diseases initially recruited from the Seoul metropolitan area, we aimed to monitor all 142 individuals during both sampling periods. However, due to the long-term nature of the study, some participants withdrew, and data collection from others was incomplete due to issues such as missing GPS data, incomplete time activity diaries, or the identification of participants who were not adults. As a result, 93 adults aged 19 and older, who had no mobility restrictions (Table 1) and no difficulties in collecting GPS data, were selected for monitoring. This number (142 adults) represents the initial pool of participants recruited for the study, not the total allergic population in Seoul.

To ensure the reliability of the data, we conducted monitoring during two separate periods: November 2022 to April 2023 and November 2023 to April 2024. These periods were chosen as they encompass the spring and winter seasons when PM_2.5_ concentrations are typically high in South Korea [23,24]. This approach allowed us to maintain consistent data collection from the selected participants.

This study was conducted following approval from the institutional review board (IRB) prior to its commencement, with details aligned with the project titled “Development of Technology to Investigate the Impact of Allergic Diseases Due to Indoor Air Pollutants from Fine Dust Infiltration” (IRB No. 2022GR0384).

**Figure A1:**
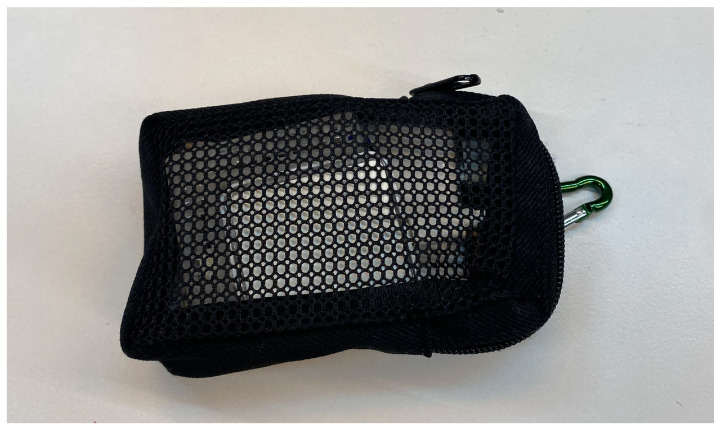
Device (PMM-130) for personal PM_2.5._

Our research team collected data from the study participants, including personal PM_2.5_ concentrations (measured using PMM-130, Brilliant & Company Co., Ltd., Seoul, Republic of Korea), indoor pollutant concentrations (measured using IAQ-C7, K-Weather Co., Ltd., Seoul, Republic of Korea) in individual residences, latitude and longitude coordinates, TAD, and survey responses. Additionally, meteorological data were gathered based on the latitude and longitude coordinates. Detailed information on data collection is described in prior studies [25] and Appendix A.1 (Table A1, Table A2 and Table A3, Figure A1). For latitude and longitude coordinates, a research application developed by our team to collect clinical data from participants was installed on the participants’ mobile phones, enabling real-time tracking of their locations. Meanwhile, personal PM_2.5_ concentrations and GPS coordinates were collected separately; if data reception failed from either device, the data could not be used, and only time periods with complete data from all sources were included in the analysis. In cases where data for less than 24 h were lost, daily average concentrations could not be directly calculated. Instead of interpolating concentrations, we estimated daily average personal PM_2.5_ exposure concentrations using TAD data and the time-weighted average (TWA) technique. The formula for calculating daily average PM_2.5_ concentrations is presented in Equation (1).

(1) $${Daily\;PM}_{2.5}=\frac{\sum_{n=1}^{i}{(C}_{i}\times T_{i})}{\sum_{n=1}^{i}T_{i}}$$

where ${Daily\;PM}_{2.5}$ represents the daily average personal PM_2.5_ exposure concentration based on the TWA technique, $C_{i}$ denotes the average PM_2.5_ concentration (µg/m^3^) in the space i, and $T_{i}$ indicates the occupancy time (h) in the space i. Unlike the conventional TWA technique, which typically does not reflect temporal characteristics and treats values in the same space as having the same dimension when the space i is measured, this study considered a space as distinct if it changed midway or occurred on a different date, even if it was physically the same location. For example, based on TAD results, if a participant’s schedule was as follows: 0:00–7:00 at home, 7:00–8:30 on public transportation, 8:30–12:00 at the office, 12:00–13:00 at a restaurant (other indoor environment), 13:00–18:00 at the office, 18:00–19:30 on public transportation, and 19:30–7:00 the next day at home, the number of spaces was not considered as 4 (home, public transportation, office, other indoor) but as 8 (home—morning, public transportation—morning, office—morning, other indoor, office—afternoon, public transportation—afternoon, home—evening, home—early morning).

For the survey, a research application was utilized, and the time activity diary centered on occupied spaces (outdoor environment, residential environment, office, educational facility, transportation, other indoor environment) was collected in 30 min intervals. Additionally, information such as the use of air purifiers in occupied spaces was surveyed on a daily basis. Other personal information, including the participants’ gender, age, weight, body mass index (BMI), and smoking status, as well as investigations into allergic diseases, were conducted at the beginning of the study.

### 2.2. Statistical Analysis

This study utilized descriptive statistics and LMEs to analyze the factors influencing daily PM_2.5_ exposure in adults. The primary objective of the study was not to predict exposure levels but to identify key determinants affecting PM_2.5_ exposure and propose strategies for exposure reduction, for which LMEs were employed. In the LMEs analysis, where personal PM_2.5_ exposure concentration was set as the dependent variable, the correlation between repeatedly measured data within the same individual could be accounted for, enabling the estimation of both intra-subject and inter-subject variance [26]. Accordingly, LMEs have been widely used in studies that identify factors correlated with personal exposure concentrations and suggest methods to reduce personal exposure [21,26]. The equation for the mixed-effects model is presented in Equation (2) below.

(2) $${log(Daily\;PM}_{2.5,ijk})=\alpha +u_{ij}+v_{ij}+\beta X_{ijk}+\varepsilon_{ijk}$$

where ${log(Daily\;PM}_{2.5,ijk})$ is the log-transformed daily PM_2.5_ exposure concentration for the k-th measurement (monitored dates for individual) on the i-th type of day (weekday/weekend) for the j-th individual. α denotes the fixed intercept, representing the average log exposure across all participants. $u_{ij}$ represents the random intercept term for each individual, capturing between-subject variability, $v_{ij}$ is the random intercept nested within individuals by type of day (weekday or weekend), capturing intra-subject variability related to day type. β represents fixed-effect coefficients for each independent variable (determinants). $X_{ijk}$ represents the values of the independent variables for each individual, type of day, and measurement occasion. $\varepsilon_{ijk}$ is the residual error, representing unexplained variation within individuals.

Daily PM_2.5_ exposure values were log-transformed to ensure normality and stabilize variance, allowing results to be interpreted as percentage changes in exposure concentrations. The model initially included variables selected from prior research as potential determinants of personal PM_2.5_ exposure, including meteorological variables (temperature, relative humidity, wind direction, wind speed, precipitation), smoking status, outdoor air pollutant (PM_10_, PM_2.5_, NO_2_, SO_2_, O_3_, CO) concentrations, measurement day type (weekday/weekend), air purifier use (home, office, other microenvironments), indoor pollutant concentrations, and type of allergic disease (asthma, rhinitis, conjunctivitis).

Due to the large number of variables initially included (Akaike information criterion: 38,280), we performed preliminary correlation analyses to streamline variable selection. For ordinal and continuous variables such as age and BMI, Spearman correlation analysis was conducted, whereas for binary variables (e.g., smoking status), point-biserial correlation analysis was employed [27]. Variables showing statistically significant correlations (*p* < 0.05) were preferentially included in the mixed-effects model. Additionally, some variables, though not significant in preliminary analyses but considered critical based on previous studies, were retained to avoid omission of relevant determinants.

The final mixed-effects model was implemented using the lmer and lmerTest packages in R statistical software (version 4.2.1) [28]. Multicollinearity among variables was analyzed using the variance inflation factor (VIF), and all variables in the final model had a VIF below 10 (Table A4). Statistically significant variables were defined as those with a *p*-value less than 0.05.

To interpret the results, effect estimates (β) obtained from the LMEs analysis were converted into percentage changes using the following equation (Equation (3)):

(3) $$Percentage\;change\;\left(\%\right)=\left(e^{\beta }-1\right)\times 100(\%)$$

where Percentage change (%) represents the percentage increase or decrease in the daily PM_2.5_ exposure for each unit change in the independent variable. β denotes fixed-effect coefficients for each independent variable (determinants) obtained from the LMEs analysis, reflecting the magnitude and direction of the relationship between each determinant and personal PM_2.5_ exposure. This interpretation method allows a clear understanding of how changes in determinants influence personal exposure concentrations, supporting the formulation of practical exposure reduction strategies.

## 3. Results

### 3.1. PM_2.5_ Exposure Characteristics of Participants

A total of 93 adults residing in the Seoul metropolitan area, diagnosed with three types of allergic diseases, were monitored for an average of approximately 164 days each, about 70% occurred on weekdays and 30% on weekends. The characteristics of the study participants and their individual exposure profiles are summarized in Table 2.

**Figure 1:**
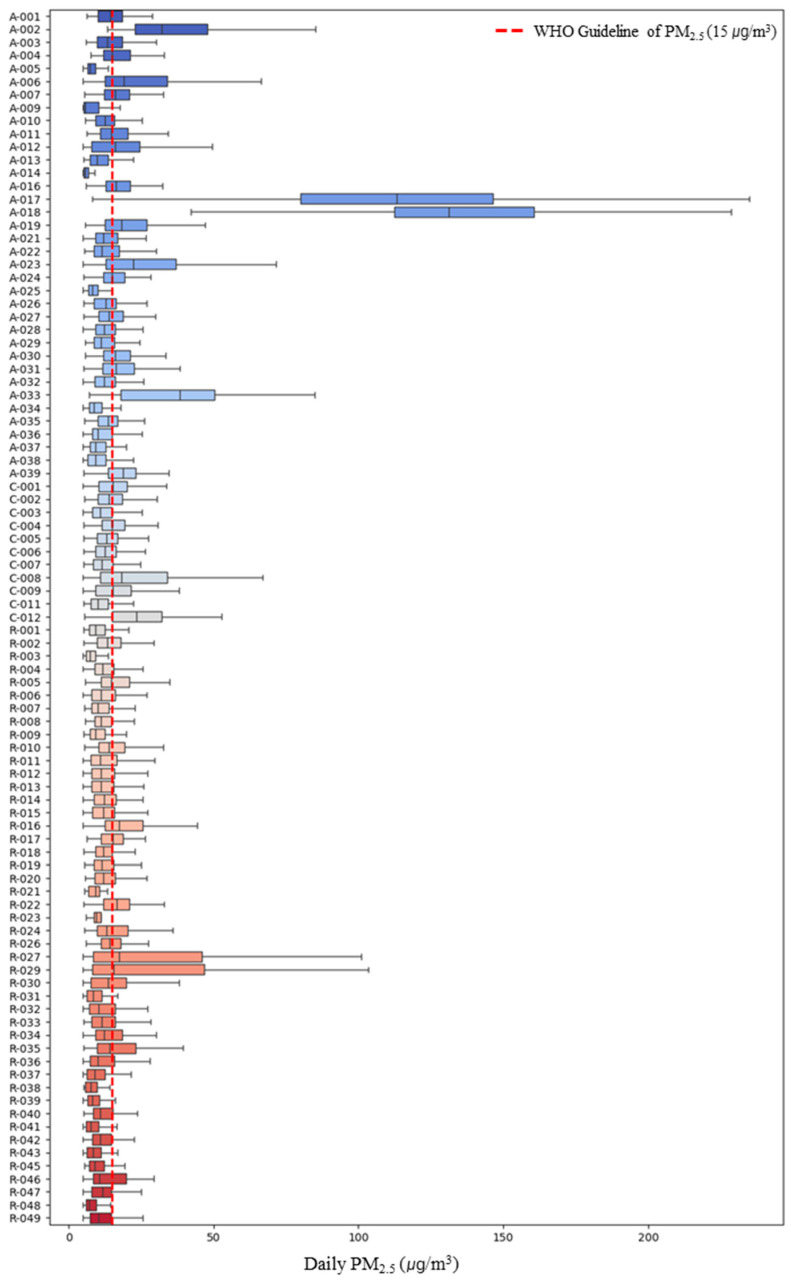
Personal PM_2.5_ exposure concentration of study participants. A (Asthma), R (Rhinitis), C (Conjunctivitis).

The personal PM_2.5_ exposure concentration of the study participants was found to be 17.38 μg/m^3^, with personal exposure concentrations on weekends (17.42 μg/m^3^) being higher than on weekdays (17.36 μg/m^3^). However, the difference in mean concentrations between weekdays and weekends was not statistically significant (*p* > 0.05). Upon examining the personal PM_2.5_ exposure concentrations of the study participants (Figure 1), a wide variation in concentrations between participants was observed. Among asthma patients, participant *A-018* exhibited the highest personal exposure concentration at 138.29 ± 46.00 μg/m^3^, the highest among all participants. Additionally, the number of days exceeding the current 24-h PM_2.5_ exposure health guideline of 15 μg/m^3^ of WHO was 6790 out of the total monitored period of 19,162 days across all participants [30], accounting for approximately 35%. Furthermore, 40 out of the 93 participants (representing 43%) had daily average concentrations exceeding the WHO guideline.

**Figure 2:**
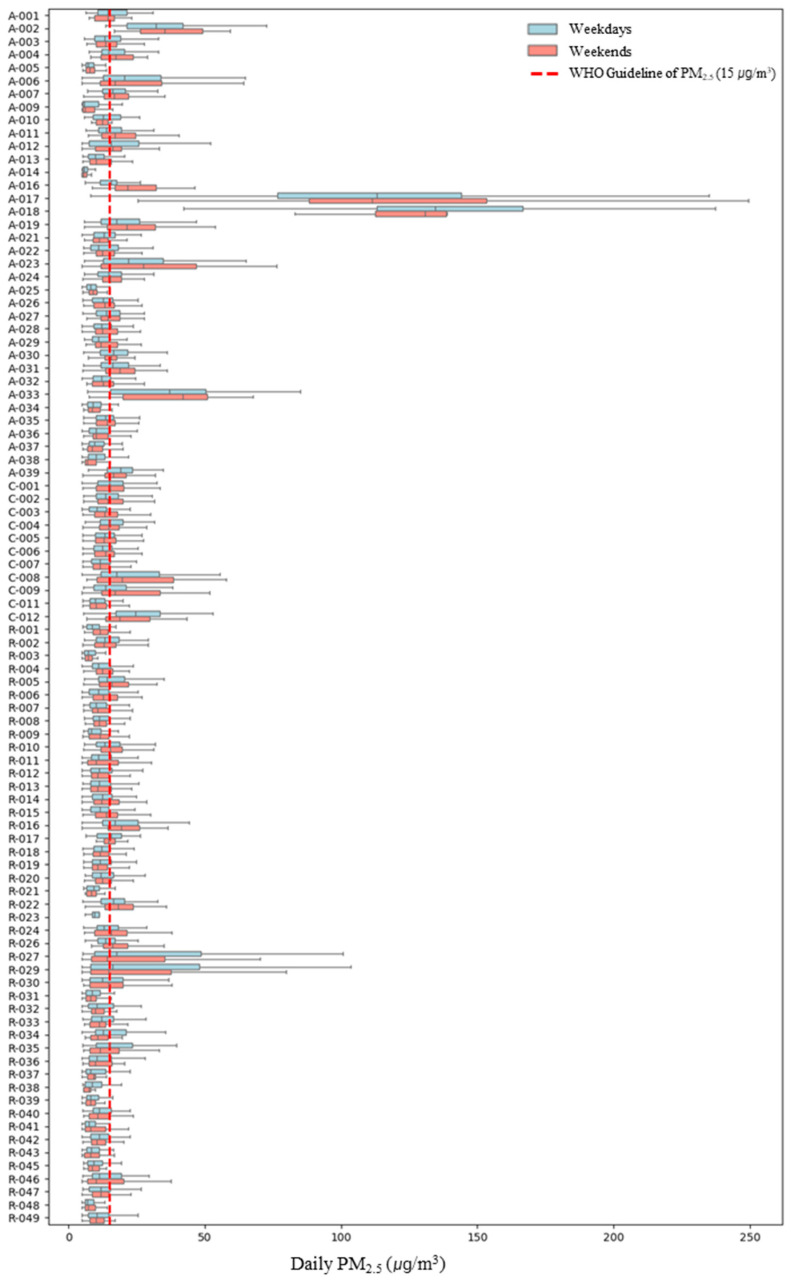
Personal PM_2.5_ exposure concentration by microenvironment on weekdays/weekdays. A (Asthma), R (Rhinitis), C (Conjunctivitis).

Upon examining personal PM_2.5_ exposure concentrations by weekday and weekend (Figure 2), it was found that personal PM_2.5_ exposure concentrations varied between weekdays and weekends depending on the participant. For 39 participants, weekday concentrations were higher, while for 54 participants, weekend personal exposure concentrations were higher. For participant *A-018*, who exhibited the highest personal exposure concentration, weekday and weekend concentrations were 139.60 ± 47.71 μg/m^3^ and 135.00 ± 42.29 μg/m^3^, respectively, indicating higher weekday concentrations but with no significant difference. In the case of *R-032*, the largest difference between weekday and weekend concentrations was observed, with weekday concentrations at 32.08 ± 74.12 μg/m^3^ and weekend concentrations at 11.26 ± 5.29 μg/m^3^, showing weekday concentrations approximately 2.9 times higher than those on weekends. Conversely, for *A-016*, weekday concentrations were 15.75 ± 5.29 μg/m^3^ and weekend concentrations were 25.39 ± 11.37 μg/m^3^, indicating that weekend exposure concentrations were approximately 1.5 times higher than those on weekdays. Therefore, to account for these individual differences, we considered weekday/weekend as a variable in the linear mixed-effects model.

Consequently, an analysis of personal PM_2.5_ exposure concentrations across six microenvironments on weekdays and weekends revealed that, when at the workplace, weekend concentrations were higher at 18.14 ± 27.36 μg/m^3^ compared to weekdays (Table 3). However, the differences in personal exposure concentrations between weekdays and weekends in microenvironments other than the workplace were not statistically significant (*p* > 0.05). Previous studies have suggested that ventilation in office buildings can significantly affect indoor PM_2.5_ exposure levels, and this impact may vary depending on the operational conditions of air conditioning systems [31]. Given this context, the difference in personal PM_2.5_ exposure concentrations between weekdays and weekends at the workplace may be influenced by changes in ventilation patterns or reduced operation of air conditioning systems on weekends compared to weekdays, leading to relatively higher personal PM_2.5_ exposure concentrations in the workplace. However, as information on specific system operation was limited in this study, further research is necessary to confirm these effects.

### 3.2. Identification of Variables Influencing Personal Exposure Concentrations

**Figure A2:**
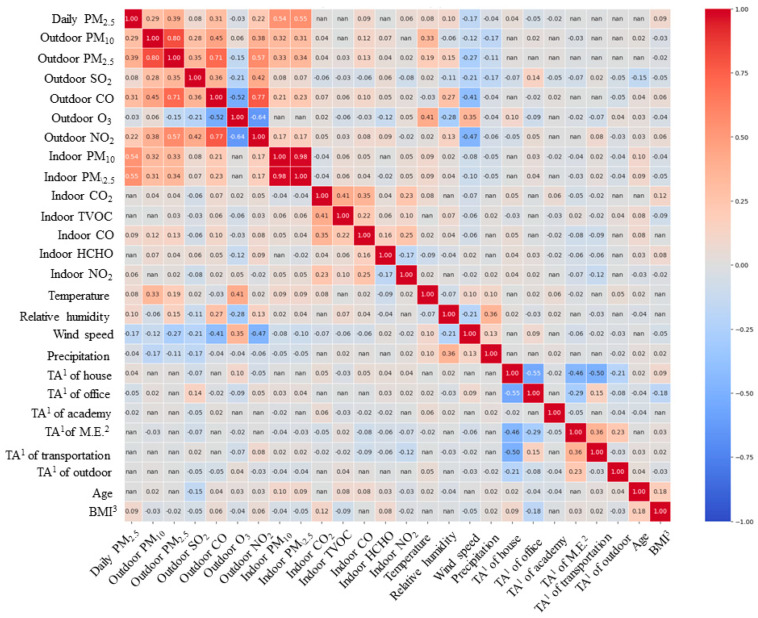
Results of Spearman correlation analysis for continuous variables. ^1^ Time activity. ^2^ Microenvironments. ^3^ Body mass index.

In this study, prior to quantitatively evaluating the extent to which variables affect personal exposure concentrations using a mixed-effects model, we first aimed to determine whether there was a correlation between personal exposure concentrations and variables through correlation analysis. Initially, Spearman correlation analysis was performed for continuous variables (Table A5), with age and BMI analyzed together, considering their ordinal variable characteristics. The correlation analysis results indicated that indoor residential CO_2_, total volatile organic compounds (TVOC), and HCHO concentrations, as well as time spent in other microenvironments (excluding home, office, educational facilities, public transportation, and outdoors), public transportation, and outdoor environments, along with age, showed no statistically significant correlation with personal exposure concentrations. In contrast, variables with statistically significant correlations included indoor residential PM_10_ and PM_2.5_ concentrations (r = 0.54, r = 0.55), which exhibited a high correlation with personal exposure concentrations. Additionally, outdoor air pollutants such as PM_2.5_ (r = 0.39), PM_10_ (r = 0.29), CO (r = 0.31), and NO_2_ (r = 0.22) in nearby outdoor air also demonstrated relatively high levels of correlation. For most other variables, the correlation with daily average concentrations was found to be somewhat low. Meanwhile, through correlation analysis among the target variables, variables potentially causing multicollinearity issues were identified first (Figure A2). The correlation coefficient between indoor residential PM_10_ and PM_2.5_ concentrations was 0.98, raising concerns about multicollinearity errors. Consequently, among these two variables, only indoor PM_2.5_ concentration, which showed a higher correlation with personal exposure concentration, was selected as an input variable.

Next, the results of the point-biserial correlation analysis between bivariate variables and personal PM_2.5_ exposure concentrations (Table 4) showed that smoking status had the highest positive correlation with a coefficient of 0.33. The correlation coefficients for gender and the presence of asthma were 0.11 and 0.12, respectively, indicating a relatively higher positive correlation compared to other variables (*p* < 0.05).

In other words, the variables ultimately used in the mixed-effects model analysis consisted of all the original target variables except for indoor residential CO_2_, TVOC, and HCHO concentrations, time spent in other indoor environments, public transportation, and outdoor environments, age, conjunctivitis, and air purifier use in the office.

### 3.3. Result of Linear Mixed-Effects Model

The LMEs was calculated using the variables selected through the prior correlation analysis. First, the daily personal PM_2.5_ exposure concentration was found to be approximately 5.80 ± 1.31 μg/m^3^ when all variables considered in this study were at their baseline levels.

It was confirmed that the variation between individuals could be as high as approximately 43.5% (Table 5), indicating that personal exposure concentrations differ significantly across individuals. This high inter-individual variability suggests that personal characteristics such as occupation, mobility patterns, and indoor environments play a critical role in determining PM_2.5_ exposure levels. In contrast, for the same individual, the difference between weekdays and weekends was approximately 5.3%, which is relatively small compared to the inter-individual variation.

These findings imply that personal exposure concentrations are more influenced by individual-specific factors rather than temporal patterns (weekdays vs. weekends). This could be attributed to fixed patterns in personal activities or consistent exposure environments regardless of the day type, such as home and workplace environments that remain relatively unchanged between weekdays and weekends.

Therefore, the variability in personal PM_2.5_ exposure is predominantly driven by differences between individuals rather than differences between weekdays and weekends. This highlights the importance of developing personalized exposure management strategies that take into account individual behaviors, activity patterns, and microenvironmental characteristics. A one-size-fits-all approach may not be effective in reducing personal exposure, emphasizing the need for tailored intervention strategies.

Upon examining the determinants of fixed effects (Table 6), the factors statistically significantly influencing personal exposure concentrations were identified as smoking status, outdoor meteorological variables (temperature, relative humidity, wind speed, precipitation), outdoor air pollutants (PM_10_, PM_2.5_, O_3_, CO), indoor PM_2.5_ and CO concentrations, time spent at home, and the operation of air purifiers in the residence (*p* < 0.05).

Among these, smoking (90.81%) was identified as a factor increasing personal PM_2.5_ exposure concentrations based on individual characteristics. Regarding meteorological variables, an increase in temperature and relative humidity was associated with increases in personal exposure concentrations of 0.29% and 0.12%, respectively. Conversely, increases in wind speed and precipitation were associated with reductions in personal exposure concentrations of 2.87% and 91.23%, respectively, showing a greater decrease compared to the effects of temperature and humidity. Among outdoor pollutants, an increase in the concentrations of gaseous substances (excluding PM_2.5_ and SO_2_) was found to increase daily personal PM_2.5_ exposure concentrations. For O_3_, a 1 ppm increase resulted in a 1231.02% increase; however, considering that the unit is ‘*ppm*’, and the average level was 0.02 ± 0.01 ppm, an increase by one standard deviation (0.01 ppm) would correspond to an estimated 1.23% increase. In contrast, unlike other air pollutants, an increase in outdoor PM_10_ concentration was associated with a 0.20% decrease in personal exposure concentration. This suggests that deciding outdoor activities based on PM_2.5_ concentration levels rather than PM_10_ in daily life may positively contribute to reducing personal exposure concentrations.

Among variables related to the indoor environment, an increase in indoor CO concentration was found to increase personal PM_2.5_ exposure concentration by 0.68% (95% CI, 0.23–1.13), while indoor PM_2.5_ concentration did not significantly affect personal exposure concentration. Regarding occupancy rates by indoor space, an increase in time spent at home was associated with a 0.19% (95% CI, 0.04–0.35) increase in personal PM_2.5_ exposure concentration, indicating the need for voluntary indoor air quality management by occupants in residential settings. In particular, an increase in CO concentration was associated with higher personal exposure concentrations, suggesting that intensive management through natural or mechanical ventilation is necessary during cooking activities (especially gas stove use), a major source of indoor CO [32]. Meanwhile, operating an air purifier in the residence was found to reduce personal exposure concentration by 5.53% (95% CI, −8.47 to −2.49), confirming that air purifier use not only improves indoor air quality within the space but also has a positive effect on reducing individual exposure levels.

## 4. Discussion

PM_2.5_ is a major risk factor for various diseases, including respiratory diseases, cancer, and cardiovascular diseases. Reducing PM_2.5_ exposure is essential for preventing these diseases. Accordingly, this study aimed to identify the factors influencing personal PM_2.5_ exposure among adults with allergic diseases in Seoul, a representative urban area in South Korea, using an LMEs model.

First, the personal PM_2.5_ exposure concentration of the study participants with allergic diseases was found to be 17.38 μg/m^3^, which exceeds the current WHO daily average PM_2.5_ guideline of 15.00 μg/m^3^ [30]. Considering that the measurement period focused on winter and spring, when high PM_2.5_ concentrations are typically observed in South Korea, the actual exposure levels may be lower. According to Lee et al. [33], the personal PM_2.5_ exposure concentration during a similar heating season for urban residents in China was 108 μg/m^3^, approximately 6.2 times higher than the levels observed in this study. A systematic literature review assessing personal exposure concentrations by national income classification [34] found that the median personal exposure concentration in high-income countries, 18.9 μg/m^3^, is similar to the levels observed in this study. Thus, compared to global personal exposure concentrations, the allergic disease patients in this study were exposed to relatively low levels of PM_2.5_.

According to Lim et al. [13], personal PM_2.5_ exposure levels can be influenced by the microenvironments that individuals occupy. Additionally, NIER [35] reports that Koreans exhibit significantly different occupancy patterns between weekdays and weekends. Specifically, individuals spend an average of 4.70 ± 4.44 h at the workplace on weekdays, but only 1.91 ± 3.46 h on weekends, representing a 2–3-fold difference. In contrast, they spend an average of 13.41 ± 4.45 h in residential environments on weekdays, increasing to 15.93 ± 4.85 h on weekends, indicating more time spent at home on weekends compared to weekdays. Consequently, an analysis of personal exposure concentrations on weekdays versus weekends revealed that 58% (54 out of 93) of the study participants had higher personal exposure concentrations on weekends compared to weekdays. However, this pattern was not consistent across all individuals. For example, participant *A-018* showed little difference, with weekday and weekend concentrations of 139.60 ± 47.71 μg/m^3^ and 135.00 ± 42.29 μg/m^3^, respectively. In contrast, participant *R-032* exhibited a weekday concentration of 32.08 ± 74.12 μg/m^3^, approximately 2.9 times higher than their weekend concentration. Prior studies [36,37] suggest that exposure levels can vary significantly depending on the spaces individuals occupy or their travel routes, with outdoor traffic pollution or indoor activities (e.g., cooking, smoking) influencing concentrations depending on the time of day. These findings indicate that the study participants’ activity patterns and microenvironment choices play a significant role in the differences in exposure between weekdays and weekends. Furthermore, the substantial inter-individual variation reflects differences in exposure due to travel routes and lifestyle habits, emphasizing the need for personalized exposure management strategies.

To identify factors influencing daily personal PM_2.5_ exposure concentrations, an LMEs model was employed, with personal PM_2.5_ exposure concentration set as the dependent variable. Participant characteristics and weekday/weekend distinctions were treated as random variables, while data from surveys or indoor/outdoor pollutant measurements based on travel routes were set as fixed variables. The analysis revealed that the difference in daily average exposure levels between individuals was approximately 43.5%, whereas the difference between weekdays and weekends for the same individual was approximately 5.3%. These results align with findings from prior studies monitoring personal exposure [38,39]. This finding indicates that individual-specific factors are the primary determinants of personal PM_2.5_ exposure rather than temporal variations. Therefore, personalized exposure reduction strategies are crucial, focusing on individual behavioral patterns, indoor air quality management, and optimized travel routes.

An analysis of the fixed-effect determinants influencing the increase in personal PM_2.5_ exposure concentrations revealed that smoking status, temperature, relative humidity, outdoor air pollutants (PM_2.5_, O_3_, CO), indoor PM_2.5_ and CO concentrations, and time spent indoors at home were significant factors. Notably, the personal exposure concentration of smokers was approximately 90.81% higher than that of non-smokers, making it the most substantial contributor. The higher personal exposure concentrations among smokers align with findings from prior studies [40,41]. Therefore, targeted smoking cessation policies and improved indoor air quality management should be prioritized to reduce exposure, especially for allergic disease patients. The confirmation that outdoor PM_2.5_ concentrations contribute to individual PM_2.5_ exposure levels reinforces the scientific basis of numerous epidemiological studies evaluating health impacts due to outdoor PM_2.5_ exposure [42]. Additionally, gaseous pollutants such as O_3_ and CO were also found to influence increases in personal exposure concentrations. O_3_ can contribute to secondary PM_2.5_ formation through photochemical reactions in the atmosphere [43,44], while CO can be generated through incomplete combustion during fuel burning in motorcycles or diesel vehicles [45,46]. Given that the Seoul metropolitan area, the primary activity region of the study participants, is characterized by dense traffic activity, it is inferred that using routes separated from congested traffic roads, rather than those adjacent to vehicle roadways, could be positively impacting and reducing PM_2.5_ exposure levels for individuals residing in urban areas. However, as the study period did not include summer months, when high sunlight duration and solar radiation could lead to elevated O_3_ concentrations, further research is deemed necessary to assess personal PM_2.5_ exposure levels and establish management strategies in response to increased O_3_ generation.

Regarding indoor environment determinants, CO concentration and time spent indoors at home were identified as primarily influencing increases in personal PM_2.5_ exposure concentrations. This result differs from studies that found that high indoor PM_2.5_ concentrations significantly affect personal exposure increases [47,48]. In South Korea, most households use liquefied natural gas or liquefied petroleum gas as primary fuel sources rather than solid fuels like coal or wood, which emit high PM_2.5_ concentrations during combustion. Additionally, the study participants’ efforts to minimize PM_2.5_ exposure, such as operating air purifiers (used during approximately 30% of the total measurement period), likely contributed to this outcome.

Conversely, an analysis of determinants reducing personal PM_2.5_ exposure concentrations revealed that outdoor environmental variables, particularly rainfall (with a reduction of 91.23% during high precipitation), were the most effective in decreasing personal exposure. Operating air purifiers in residential settings was also found to have a positive effect on reducing personal PM_2.5_ exposure concentrations compared to other spaces. Fujino et al. [49] confirmed that atmospheric PM_2.5_ concentrations decrease significantly with precipitation, and Tai et al. [50] and Chate et al. [51,52] reported a strong negative correlation between rainfall and atmospheric pollutants. In this study, correlation analysis between variables also showed negative correlation coefficients of −0.11 and −0.27 between precipitation and wind speed with outdoor PM_2.5_, respectively (*p* < 0.05). Thus, it was confirmed that reductions in outdoor PM_2.5_ concentrations due to climatic conditions such as rainfall led to decreased personal exposure levels, demonstrating that national fine dust reduction policies aimed at lowering outdoor PM_2.5_ are ultimately effective in reducing individual PM_2.5_ exposure. Furthermore, operating air purifiers in homes was confirmed to be effective in reducing personal exposure levels, interpreted as a result of the air purifiers’ filtering technology effectively removing indoor PM_2.5_, thereby reducing particulate matter generated indoors or infiltrated from outside. Given that the study participants were adults with allergic diseases (asthma, rhinitis, conjunctivitis), this suggests that air purifier use could play a critical role in preventing the exacerbation of respiratory symptoms by improving indoor air quality. Prior studies have reported that air purifiers can reduce indoor particulate matter concentrations by up to 61.8% [53], and numerous studies have investigated the effectiveness of air purifiers in reducing PM_2.5_ exposure [54,55,56]. These findings indicate that activities aimed at reducing PM_2.5_ concentrations in the air, such as operating indoor air purifiers, are ultimately effective in lowering personal PM_2.5_ exposure levels.

Meanwhile, in the case of allergic diseases, no differences in personal exposure concentrations were observed based on the type of disease. This suggests that the exposure patterns of individuals with allergic diseases may be more significantly influenced by external factors—such as activity patterns, outdoor air quality, or the use of air purifiers in residences—rather than the specific characteristics of the disease itself. Therefore, it is considered that PM_2.5_ exposure management strategies for individuals with allergic diseases may be more effective when focused on commonly applicable environmental factors (e.g., indoor air quality management, optimization of travel routes) rather than being tailored to specific disease types. However, a limitation of this study is that it did not further analyze the correlation between the severity of symptom manifestation by disease type and exposure concentrations. Additionally, the study did not include a non-allergic comparison group, which limits the ability to directly compare PM_2.5_ exposure between allergic and non-allergic individuals in the same area. Future research is expected to contribute practically to disease management by exploring in detail the impact of PM_2.5_ exposure on the exacerbation of allergic symptoms, as well as by incorporating non-allergic cohorts to better understand exposure differences.

## 5. Conclusions

This study analyzed the factors influencing personal PM_2.5_ exposure levels among adults with allergic diseases in Seoul, a representative urban area in South Korea, using an LMEs model. The primary objective was to identify key factors contributing to personal exposure and to propose effective reduction strategies based on individual characteristics and environmental variables.

The measurement results revealed that the personal PM_2.5_ exposure concentration of the study participants was 17.38 μg/m^3^, exceeding the WHO daily recommended guideline of 15.00 μg/m^3^. However, when compared globally, the exposure levels in this study were found to be relatively low. Meanwhile, PM_2.5_ exposure concentrations varied among the study participants, with significant differences observed depending on the microenvironments that they occupied.

The LMEs model identified several key factors contributing to increased personal PM_2.5_ exposure, including smoking status (with smokers showing 90.81% higher exposure than non-smokers), temperature, relative humidity, outdoor pollutants (PM_2.5_, O_3_, CO), indoor PM_2.5_ and CO concentrations, and time spent in residential environments.

Conversely, factors that significantly reduced exposure included rainfall (with a reduction of 91.2%), wind speed, and the use of air purifiers in residential settings. These results suggest that environmental improvements (e.g., reducing outdoor PM_2.5_ through policy) and individual efforts (e.g., using air purifiers) are both essential for reducing personal exposure.

Furthermore, these findings indicate that individual activity patterns, microenvironments, and lifestyle habits play a critical role in determining PM_2.5_ exposure levels. The inter-individual variation in exposure was approximately 43.5%, while the difference between weekdays and weekends for the same individual was about 5.3%. This underscores the need for personalized exposure reduction strategies. Additionally, the effectiveness of outdoor air quality improvements, rainfall, and air purifier use in reducing exposure reaffirms the importance of both national fine dust reduction policies and individual efforts to improve indoor air quality. While this study revealed that PM_2.5_ exposure patterns in allergic disease patients are more influenced by environmental factors and behavioral patterns than by disease type, it has a limitation in not analyzing the correlation between exposure concentrations and symptom manifestation by disease type. Future research should explore in detail the impact of PM_2.5_ exposure on the exacerbation of allergic symptoms to identify practical contributions to disease management.

By analyzing personal PM_2.5_ exposure levels and their influencing factors using the LMEs model, this study provides scientific evidence for personalized exposure reduction strategies. These findings offer practical insights for reducing health risks associated with PM_2.5_, particularly for vulnerable populations such as individuals with allergic diseases.

## Figures and Tables

**Table 1:** Participant information for this study.

Item		Information
Sampling date		1st: 21 November 2022–16 April 2023 2nd: 1 November 2023–16 April 2024
Sex	Male	30
	Female	63
Age (median, range)		39.94 (38, 19–66)

**Table 2:** Characteristics of participants.

Item ^1^				Unit	All	Weekdays	Weekends
Demographics	Number of participants (%)			-	93	93	
	Number of days monitored (S.D.)			Days/person	164.60	118.20 (71.81)	46.40 (28.19)
	Gender (%)	Male		-	30	31 (32.26)	
		Female		-	63	63 (67.74)	
	Smoke (%)	Smoke		-	90	90 (96.77)	
		Non-smoke		-	3	3 (3.23)	
	Weight (S.D.)			kg		64.31(12.68)	
	Ages (%)	19–29		-	26	26 (27.96)	
		30–39		-	23	23 (24.73)	
		40–49		-	15	15 (16.13)	
		50–59		-	19	19 (20.43)	
		60–65		-	10	10 (10.75)	
	BMI ^2^	Underweight		kg/m^2^	63	63 (67.74)	
		Normal weight		kg/m^2^	15	15 (16.13)	
		Overweight		kg/m^2^	8	8 (8.60)	
		Obesity class I		kg/m^2^	4	4 (4.30)	
		Obesity class II		kg/m^2^	2	2 (2.15)	
		Obesity class III		kg/m^2^	1	1 (1.08)	
Environmental factors	Temperature (S.D.)			°C	4.26	4.27 (6.75)	4.23 (6.67)
	Relative humidity (S.D.)			%	1.71	60.00 (16.08)	58.85 (16.44)
	Wind speed (S.D.)			m/s	59.68	1.73 (0.88)	1.65 (0.85)
	Precipitation (S.D.)			mm	0.00	0.00 (0.00)	0.00 (0.01)
	Outdoor PM_10_ (S.D.)			μg/m^3^	46.30	46.24 (32.83)	46.47 (27.03)
	Outdoor PM_2.5_ (S.D.)			μg/m^3^	24.24	24.09 (14.98)	24.66 (15.53)
	Outdoor NO_2_ (S.D.)			ppm	0.02	0.03 (0.01)	0.02 (0.01)
	Outdoor SO_2_ (S.D.)			ppm	0.00	0.00 (0.00)	0.00 (0.00)
	Outdoor O_3_ (S.D.)			ppm	0.02	0.02 (0.01)	0.03 (0.01)
	Outdoor CO (S.D.)			ppm	0.51	0.51 (0.17)	0.50 (0.15)
	Personal PM_2.5_ (S.D.)			μg/m^3^	17.38	17.36 (24.52)	17.42 (21.24)
	PM_10_ in house (S.D.)			μg/m^3^	49.59	49.47 (356.93)	49.91 (358.38)
	PM_2.5_ in house (S.D.)			μg/m^3^	34.64	34.58 (249.89)	34.80 (250.87)
	CO_2_ in house (S.D.)			ppm	877.35	866.28 (315.59)	905.43 (359.29)
	TVOC ^3^ in house (S.D.)			ppm	494.84	491.44 (720.09)	504.23 (736.07)
	CO in house (S.D.)			ppm	2.33	2.34 (2.18)	2.31 (2.19)
	HCHO in house (S.D.)			ppm	27.01	26.96 (34.95)	27.12 (34.41)
	NO_2_ in house (S.D.)			ppm	0.00	0.00 (0.00)	0.00 (0.00)
Indoor management (use air purifier)	House		Yes (%)	-	6031	4335 (71.71)	1696 (28.29)
			No (%)	-	12,679	9902 (71.88)	3587 (28.12)
	Office		Yes (%)	-	2328	1946 (70.08)	382 (29.92)
			No (%)	-	16,382	11,481 (83.59)	4901 (16.41)
	Other M.E. ^4^		Yes (%)	-	1632	1180 (72.30)	452 (27.70)
			No (%)	-	17,078	12,247 (71.71)	4831 (28.29)
Time activity pattern	House (S.D.)			h	14.98	14.19 (6.03)	16.97 (7.14)
	Office (S.D.)			h	4.10	5.28 (4.96)	1.13 (3.19)
	Academy (S.D.)			h	0.06	0.07 (0.61)	0.02 (0.33)
	Other M.E.^4^ (S.D.)			h	2.68	2.29 (4.52)	3.67 (5.96)
	Transportation (S.D.)			h	1.37	1.44 (1.81)	1.19 (1.85)
	Outdoor (S.D.)			h	0.80	0.72 (1.60)	1.01 (2.16)
Allergy disease	Asthma (%)			-	36	36 (38.71)	
	Rhinitis (%)			-	46	46 (49.46)	
	Conjunctivitis (%)			-	11	11 (11.83)	
Sampling information	Season		Spring (%)	days/person	52.58	52.58 (31.94)	
			Fall (%)	days/person	14.67	14.67 (8.91)	
			Winter (%)	days/person	97.35	97.35 (59.14)	
	Day		Monday	days/person	23.55	23.55 (14.31)	
			Tuesday	days/person	23.61	23.61 (14.34)	
			Wednesday	days/person	23.67	23.67 (14.38)	
			Thursday	days/person	23.67	23.67 (14.38)	
			Friday	days/person	23.71	23.71 (14.40)	
			Saturday	days/person	23.22	23.22 (14.11)	
			Sunday	days/person	23.18	23.18 (14.08)	

^1^ Data are n (%), Mean (S.D.). ^2^ The BMI classification referenced in this study is based on guidelines provided by Obesity standards in Korea [29]. ^3^ Total volatile organic compounds. ^4^ Microenvironments.

**Table 3:** Personal PM_2.5_ exposure concentration by microenvironments on weekdays and weekends.

Space	Weekdays/ Weekends	Personal PM_2.5_ (μg/m^3^)		*p*-Value
		Mean	S.D.	
House	Weekdays	17.04	26.39	>0.05
	Weekends	17.16	23.80	
Office	Weekdays	14.87	22.02	<0.05
	Weekends	18.14	27.36	
Academy	Weekdays	21.73	59.19	>0.05
	Weekends	13.28	9.32	
Other M.E. ^1^	Weekdays	15.31	17.79	>0.05
	Weekends	15.74	19.72	
Transportation	Weekdays	15.57	20.00	>0.05
	Weekends	15.27	21.00	
Outdoor	Weekdays	16.62	14.45	>0.05
	Weekends	16.79	15.90	

^1^ Microenvironments.

**Table 4:** The results of the point-biserial correlation analysis.

Variable		Correlation	*p*-Value
Gender	Man (1)	0.11	0.00
	Woman (0)		
Asthma	Yes (1)	0.12	0.00
	No (0)		
Rhinitis	Yes (1)	−0.03	0.00
	No (0)		
Conjunctivitis	Yes (1)	−0.01	0.31
	No (0)		
Smoke	Smoke (1)	0.33	0.00
	Non-smoke (0)		
Use air purifier in house	Yes (1)	−0.03	0.00
	No (0)		
Use air purifier in office	Yes (1)	−0.01	0.11
	No (0)		
Use air purifier in M.E. ^1^	Yes (1)	−0.02	0.00
	No (0)		

^1^ Microenvironments.

**Table 5:** Random-effects determinants from mixed-effects model for personal PM_2.5_ exposure.

Variable	Variance	S.D.
Intercept (α)	1.7575	0.2693
Intercept of ID/Week	0.0028	0.0534
Intercept of ID	0.1304	0.3611
$\mathrm{Residual}\;(\varepsilon_{ijk}$)	0.1974	0.4443

**Table 6:** Fixed-effects determinants from mixed-effects model for personal PM_2.5_ exposure.

Fixed Effects Determinants	Reference Group	Percentage Change (%)	95%CI	*p*-Value
Demographics				
Gender (man)	Woman (0)	−2.19	−1.29 to 18.53	0.82
Smoke	Yes (1), No (0)	90.81	22.02 to 198.37	0.01
BMI ^1^				
Normal weight	Underweight	22.32	−20.41 to 87.99	0.36
Overweight	Underweight	19.83	−25.79 to 93.51	0.46
Obesity class I	Underweight	30.86	−20.03 to 114.14	0.29
Obesity class II	Underweight	48.52	−23.72 to 189.17	0.25
Obesity class III	Underweight	47.29	−39.21 to 256.84	0.39
Environmental factors				
Outdoor				
Temperature	-	0.29	0.17 to 0.41	0.00
Relative humidity	-	0.12	0.08 to 0.17	0.00
Wind speed	-	−2.87	−3.73 to −2.01	0.00
Precipitation	-	−91.23	−97.31 to −71.43	0.00
Outdoor PM_10_	-	−0.20	−0.23 to −0.17	0.00
Outdoor PM_2.5_	-	1.38	1.30 to 1.46	0.00
Outdoor NO_2_	-	36.05	−59.09 to 352.40	0.62
Outdoor SO_2_	-	126,054.03	−99.45 to 29,196,639,708.61	0.26
Outdoor O_3_	-	1231.02	431.75 to 3231.65	0.00
Outdoor CO	-	22.47	12.39 to 33.46	0.00
In house				
PM_2.5_ in house	-	0.01	0.01 to 0.01	0.00
CO in house	-	0.68	0.23 to 1.13	0.00
NO_2_ in house	-	104.75	−56.83 to 871.12	0.37
Time activity pattern				
Use air purifier in house	Yes (1), No (0)	−5.53	−8.47 to −2.49	0.00
Use air purifier in M.E. ^2^	Yes (1), No (0)	3.65	−0.10 to 7.55	0.06
Allergy disease				
Asthma	Yes (1), No (0)	6.57	−13.23 to 30.87	0.55
Rhinitis	Yes (1), No (0)	3.92	−22.24 to 38.89	0.80

^1^ The BMI classification referenced in this study is based on guidelines provided by obesity standards in Korea [29]. ^2^ Microenvironments.

## Data Availability

The data presented in this study cannot be provided by the corresponding authors due to privacy policy.

## Abbreviations

The following abbreviations are used in this manuscript:

LMEs	Linear mixed-effects
WHO	World Health Organization
GPS	Global Positioning System
TAD	Time activity diary
TWA	Time-weighted average
M.E.	Microenvironments
VIF	Variance inflation factor
BMI	Body mass index
PM_2.5_	Particulate matter with an aerodynamic diameter of 2.5 μm or less
PM_10_	Particulate matter with an aerodynamic diameter of 10 μm or less
TVOC	Total volatile organic compounds

## Appendix A

### Appendix A.1. Data Collection

Our research team collected data from the study participants, including personal PM_2.5_ concentrations, indoor pollutant concentrations in residences, latitude and longitude coordinates, and information on occupied spaces through surveys and time activity diaries (Table A1). Additionally, meteorological data were collected based on the gathered latitude and longitude coordinates, utilizing data from the Automated Weather System (AWS) of the Korea Meteorological Administration’s disaster prevention observation network.

**Table A1:** Information on data collection.

Variable	Location	Data Sampling Unit	Device
Personal PM_2.5_	All	1 min	PMM-130 ^5^
Indoor	House	1 min	IAQ-C7 ^6^
GPS ^1^	All	1 min	Application
Survey	All	1 day	Application
TAD ^2^	All	1 day	Application
AWS ^3^	Outdoor	1 min	AWS
Air pollution ^4^	Outdoor	1 h	Air-Korea

^1^ Global positioning system. ^2^ Time activity diary. ^3^ Automatic weather station. ^4^ PM_10_, PM_2.5_, O_3_, NO_2_, SO_2_, CO. ^5^ PMM-130 (Brilliant & Company Co., Ltd., Seoul, Republic of Korea). ^6^ IAQ-C7 (K-Weather Co., Ltd., Seoul, Republic of Korea).

In this study, a low-cost light-scattering-based sensor device (PMM-130, IAQ-C7) was utilized to facilitate the measurement of indoor and personal PM_2.5_ exposure concentrations for the study participants, with details provided in Table A2. For personal PM_2.5_ exposure concentrations, the device was encased in a mesh pouch (Figure A1) to allow smooth measurement of PM_2.5_ in the air and was attached to a bag or outerwear at a height of approximately 1.5 m within the breathing zone, enabling participants to carry it at all times. However, low-cost sensor devices are significantly affected by external factors, such as humidity, compared to gravimetric or beta-ray methods, raising concerns about the accuracy of the measured values. To address this, in this study, the GRIMM 11-A, widely used as a reference instrument for light-scattering devices, was employed to derive a calibration equation prior to measurement, ensuring suitability for real-world environments. The calibration method was verified and detailed in a previous study [57].

**Table A2:** Specifications of the measurement devices for PM_2.5_ used in the study.

Items	PMM-130	IAQ-C7
Device		
	48 (W) × 21 (H) × 48 (D) mm, 0.45 kg	200 (W) × 120 (H) × 50 (D) mm, 0.75 kg
Metrics	PM_2.5_, PM_10_, temperature, relative humidity	PM_10_, PM_2.5_, CO_2_, TVOC, HCHO, NO_2_, CO, temperature, relative humidity
Measurement range	0–1000 µg/m^3^ (PM_2.5_)	0–1000 µg/m^3^ (PM_2.5_)
Flow rate	-	0.1 L/min
Operating temperature	−10–+60 °C	−5~+60 °C
Supply voltage	5 V 2 A DC in/≤200 mA/h	-
Performance grades ^1^	1st grade (86.2%)	1st grade (90.7%)
Communication	Wi-Fi, Bluetooth	WiFi/LTE/LTEM Module
Data storage	-	Micro SD/Server
Measurement range	0–1000 µg/m^3^ (PM_2.5_)	0–1000 µg/m^3^ (PM_2.5_)

^1^ Results of the performance certification evaluation for PM_2.5_ portable measurement devices conducted by the Ministry of Environment in Korea.

**Table A3:** The location of automatic weather station (AWS) and Air-Korea.

Type of Sampling Site	Site Name	Latitude–Longitude Code
AWS ^1^	Gangnam	37°49′82.00″ N, 127°08′16.20″ E
	Gangbuk	37°63′97.20″ N, 127°02′57.60″ E
	Gangseo	37°57′39.00″ N, 126°82′95.30″ E
	Gwangmyeong	37°47′86.40″ N, 126°86′51.92″ E
	Guro	37°49′32.80″ N, 126°82′62.90″ E
	Geumcheon	37°46′55.10″ N, 126°90′01.60″ E
	Meteorological administration	37°49′33.00″ N, 126°91′74.60″ E
	Nowon	37°62′18.60″ N, 127°09′19.20″ E
	Dongdaemoon	37°58′46.30″ N, 127°06′03.60″ E
	Mapo	37°55′16.50″ N, 126°92′91.50″ E
	Seongbuk	37°61′13.40″ E, 126°99′98.10″ E
	Yeongdeungpo	37°52′70.60″ N, 126°90′70.50″ E
	Jungnang	37°58′55.10″ N, 127°08′68.20″ E
	Hangang	37°52′48.90″ N, 126 93′90.40″ E
Air-Korea	Gangnam-gu	37°31′02.00″ N, 127°02′50.00″ E
	Gangdong-gu	37°31′48.00″ N, 127°07′26.00″ E
	Gangbuk-gu	37°38′22.00″ N, 127°01′33.00″ E
	Gangseo-gu	37°33′03.00″ N, 126°50′58.00″ E
	Gwanak-gu	37°28′42.00″ N, 126°57′05.00″ E
	Gwangjin-gu	37°32′19.00″ N, 127°04′56.00″ E
	Guro-gu	37°29′44.00″ N, 126°53′15.00″ E
	Geumcheon-gu	37°27′22.00″ N, 126°53′43.00″ E
	Nowon-gu	37°39′15.00″ N, 127°03′24.00″ E
	Dobong-gu	37°40′08.00″ N, 127°02′50.00″ E
	Dongdaemun-gu	37°34′28.00″ N, 127°02′23.00″ E
	Dongjak-gu	37°30′45.00″ N, 126°56′21.00″ E
	Mapo-gu	37°33′49.00″ N, 126°54′30.00″ E
	Seodaemun-gu	37°34′45.00″ N, 126°56′12.00″ E
	Seocho-gu	37°29′01.00″ N, 127°01′57.00″ E
	Seongdong-gu	37°33′48.00″ N, 127°02′11.00″ E
	Seongbuk-gu	37°35′20.00″ N, 127°01′00.00″ E
	Songpa-gu	37°30′52.00″ N, 127°06′21.00″ E
	Yangcheon-gu	37°31′02.00″ N, 126°52′00.00″ E
	Yeongdeungpo-gu	37°31′34.00″ N, 126°53′47.00″ E
	Yongsan-gu	37°31′57.00″ N, 126°59′24.00″ E
	Eunpyeong-gu	37°36′09.00″ N, 126°55′45.00″ E
	Jongno-gu	37°34′23.00″ N, 126°58′45.00″ E
	Jung-gu	37°33′49.00″ N, 126°59′51.00″ E
	Jungnang-gu	37°36′23.00″ N, 127°05′34.00″ E

^1^ Automatic weather station.

### Appendix A.2. Results of Statistical Analysis

**Table A4:** Result of variance inflation factor (VIF).

	Site Name
Air purifier used in M.E. ^1^	1.02
Asthma	1.52
BMI ^2^	1.55
CO in house	1.05
Outdoor CO	4.69
Occupancy time in academy	1.01
Gender	1.18
Occupancy time in house	1.34
NO_2_ in house	1.00
Outdoor NO_2_	4.76
Outdoor O_3_	2.98
Occupancy time in office	1.34
Outdoor PM_10_	1.93
Outdoor PM_2.5_	3.69
Precipitation	1.07
Relative humidity	1.33
Rhinitis	1.31
Smoke	1.13
Outdoor SO_2_	1.43
Temperature	1.54
Wind speed	1.31
Air purifier use in house	1.02
Outdoor PM_2.5_	3.69
Precipitation	1.07
Relative humidity	1.33
Rhinitis	1.31
Smoke	1.13
Outdoor SO_2_	1.43
Temperature	1.54
Wind speed	1.31
Air purifier use in house	1.02

^1^ Microenvironments. ^2^ Body mass index.

**Table A5:** The results of the Spearman correlation analysis.

Variable	Correlation	*p*-Value
Temperature	0.08	0.00
Relative humidity	0.10	0.00
Wind speed	−0.17	0.00
Precipitation	−0.04	0.00
Outdoor PM_10_	0.29	0.00
Outdoor PM_2.5_	0.39	0.00
Outdoor SO_2_	0.08	0.00
Outdoor CO	0.31	0.00
Outdoor O_3_	−0.03	0.00
Outdoor NO_2_	0.22	0.00
PM_10_ in house	0.54	0.00
PM_2.5_ in house	0.55	0.00
Outdoor SO_2_	0.08	0.00
Outdoor CO	0.31	0.00
Outdoor O_3_	−0.03	0.00
Outdoor NO_2_	0.22	0.00
PM_10_ in house	0.54	0.00
PM_2.5_ in house	0.55	0.00
CO_2_ in house	0.01	0.12
TVOC in house	0.00	0.55
CO in house	0.09	0.00
HCHO in house	0.00	0.51
NO_2_ in house	0.06	0.00
Occupancy time in house	0.04	0.00
Occupancy time in office	−0.05	0.00
Occupancy time in academy	−0.02	0.00
Occupancy time in other M.E. ^1^	−0.01	0.47
Occupancy time in transportation	−0.01	0.14
Occupancy time in outdoor	0.00	0.99
Age	0.00	0.99
BMI ^2^	0.09	0.00

^1^ Microenvironments. ^2^ The BMI classification referenced in this study is based on guidelines provided by obesity standards in Korea [29].
